# Sentinel Lymph Node Biopsy in Early Stages of Oral Squamous Cell Carcinoma Using the Receptor-Targeted Radiotracer ^99m^Tc-Tilmanocept

**DOI:** 10.3390/diagnostics11071231

**Published:** 2021-07-08

**Authors:** Christian Doll, Claudius Steffen, Holger Amthauer, Nadine Thieme, Thomas Elgeti, Kai Huang, Kilian Kreutzer, Steffen Koerdt, Max Heiland, Benedicta Beck-Broichsitter

**Affiliations:** 1 Department of Oral and Maxillofacial Surgery, Charité–Universitätsmedizin Berlin, Corporate Member of Freie Universität Berlin and Humboldt-Universität zu Berlin, Augustenburger Platz 1, 13353 Berlin, Germany; claudius.steffen@charite.de (C.S.); kilian.kreutzer@charite.de (K.K.); steffen.koerdt@charite.de (S.K.); max.heiland@charite.de (M.H.); benedicta.beck-broichsitter@charite.de (B.B.-B.); 2 Department of Nuclear Medicine, Charité–Universitätsmedizin Berlin, Corporate Member of Freie Universität Berlin and Humboldt-Universität zu Berlin, Augustenburger Platz 1, 13353 Berlin, Germany; holger.amthauer@charite.de (H.A.); kai.huang@charite.de (K.H.); 3 Department of Radiology, Charité–Universitätsmedizin Berlin, Corporate Member of Freie Universität Berlin and Humboldt-Universität zu Berlin, Augustenburger Platz 1, 13353 Berlin, Germany; nadine.thieme@charite.de; 4 Department of Radiology-Pediatric Radiology, Charité–Universitätsmedizin Berlin, Corporate Member of Freie Universität Berlin and Humboldt-Universität zu Berlin, Augustenburger Platz 1, 13353 Berlin, Germany; thomas.elgeti@charite.de

**Keywords:** oral squamous cell carcinoma, sentinel lymph node biopsy, neck dissection, radiotracer, tilmanocept

## Abstract

Neck management in patients with early-stage, clinically node-negative oral squamous cell carcinoma (OSCC) remains a matter of discussion. Sentinel lymph node biopsy (SLNB) represents a treatment alternative to avoid elective neck dissection (END) in this cohort and different protocols and tracers exist. Here we present the clinical outcome of SLNB using ^99m^Tc-tilmanocept in a two-day protocol in patients suffering from early-stage OSCC. A total of 13 patients (males: 6; females: 7; mean age: 65.7 years, ranging from 47 to 89 years) were included in this study. Most of the patients suffered from an OSCC of the floor of mouth (*n* = 6), followed by tongue (*n* = 5) and upper alveolar crest/hard palate (*n* = 2). Sentinel lymph nodes (SLNs) were successfully identified in all cases (range: 1–7). The average length of hospital stay was 4.7 days (range: 3–8 days) and mean duration of surgical intervention was 121 min (range: 74–233 min). One patient who suffered from an OSCC of the tongue was sentinel lymph node positive (SLN+). The mean follow-up for all sentinel lymph node negative (SLN-) patients (*n* = 12) was 20.3 months (range: 10–28 months). No local or nodal recurrences were observed within the observation period. In our patient cohort, SLNB using ^99m^Tc-tilmanocept in a two-day protocol proved to be a reliable and safe staging method for patients suffering from early-stage, clinically node-negative OSCC. These results and their possible superiority to colloid tracers have to be confirmed in a prospective randomized controlled study.

## 1. Introduction

Oral cancer is one of the most common cancers worldwide and has a high mortality rate [1]. Most of these malignant lesions diagnosed within the oral cavity are classified as oral squamous cell carcinomas (OSCCs) [2]. The staging process includes detection of cervical lymph node metastases, which is an important prognostic factor of this disease. Surgery forms the basis of treatment for most patients with OSCC, but there is no consistent agreement on neck management in cases without clinical signs of cervical lymph node metastasis (cN0) [3].

The procedure of elective neck dissection (END) in early-stage cN0 OSCC is under debate due to its potential comorbidities and questionable need from an oncological perspective on the overall outcome, since the incidence of cervical (occult) lymph node metastasis is only about 30% [4,5]. However, a prospective, randomized controlled trial found significantly higher rates of disease-free survival when performing an END in contrast to a watchful waiting strategy in early-stage OSCC [5]. Although END might be associated with higher disease-free survival compared to watchful waiting, it is associated with higher morbidity and increased healthcare costs [3].

Institutions and national guidelines differ on how to proceed in cases of cN0 necks, especially in early stages of OSCC. In this context, reliable and less invasive options such as sentinel lymph node biopsy (SLNB) become increasingly relevant. In countries such as the UK, Spain and the Netherlands, SLNB is accepted as the standard of care. However, clinical implementation is not homogenously performed throughout the departments [6]. According to the most current German guideline for oral cancer published in March 2021, which had been under discussion as a preliminary version since 2019, SLNB can be offered to patients with early-stage OSCC when a transcervical approach is not necessary [7].

There are several multicenter studies with comparable results defining SLNB as a secure method for staging in cN0 necks [6,8,9,10]. An extensive meta-analysis published by Liu et al. in 2017 detected a pooled sensitivity of 0.87 and a negative predictive value of 0.94 in early-stage OSCC patients receiving SLNB [11]. Recently, the results of two randomized clinical trials comparing SLNB and END have become available. Garrel et al. demonstrated the oncological equivalence of these procedures in early-stage oral and oropharyngeal cancers with better initial functional outcomes for patients who received SLNB [12]. Hasegawa et al. showed similar overall survival and disease-free survival for the END and SLNB groups in early-stage oral cancers with lower postoperative disability in the SLNB group [13].

Different tracers were used in studies with varying negative predictive values. A causal relationship between the negative predictive value and different tracers is unclear though. There are various tracers available for SLNB, but some new tracers seem to feature superior properties [14]. One novel tracer is ^99m^Tc-tilmanocept, which has been available for the indications of melanoma, breast cancer and OSCC in the European Union since 2014 [15]. It binds to the CD206 mannose receptor on macrophages and is thus taken up into the lymph nodes. Its rapid clearance at the injection site offers benefits, since lymph nodes close to the injection site are thus detected more reliably, especially in case of a floor of mouth tumor [6,16,17]. A phase III multi-institutional trial including patients with OSCC detected a high sentinel identification rate of 97.6% and a low false-negative rate of 2.56% using this novel tracer [18]. Overall, to date, only a few studies exist that describe SLNB with ^99m^Tc-tilmanocept in OSCC [17,18,19,20].

Based on the convincing results published in numerous studies we introduced SLNB in our department to avoid neck dissection in patients suffering from early-stage OSCC. Both colloids and tilmanocept are used as tracers. In the present study, we evaluated the treatment of SLNB using ^99m^Tc-tilmanocept in a two-day protocol in patients suffering from early-stage OSCC. 

## 2. Materials and Methods

### 2.1. Ethics Statement

The Ethics Committee of the Faculty of Medicine, Charité Berlin, approved this retrospective study (EA2/043/20).

### 2.2. Patients

We analyzed the data of all patients treated with primary radical tumor resection and SLNB using ^99m^Tc-tilmanocept (Lymphoseek^®^) in a two-day protocol at the Department of Oral and Maxillofacial Surgery of the Charité-Universitätsmedizin Berlin, Germany, until 31 March 2020. 

Inclusion criteria were early-stage primary OSCC (cT1/cT2) without any clinical sign of lymph node metastasis (cN0) in routinely performed preoperative computed tomography (CT) or magnetic resonance imaging (MRI), as well as clinical examination. Cervical node negativity was re-evaluated in a simple blind technique by an experienced radiologist in the CT/MRI scans of the patients. Patients who suffered from synchronous malignancies affecting the head and neck area or previous tumors within this area were excluded from the study. Patients with previous neck dissection and/or radiotherapy in the head and neck region were also excluded. Only patients with a minimum follow-up of 6 months with at least one postoperative CT/MRI scan were included. A total of 13 out of 20 OSCC patients treated with SLNB using ^99m^Tc-tilmanocept within the inclusion period met the inclusion criteria.

The primary endpoint of this study was recurrence-free survival (RFS), which was defined as the time from primary treatment to recurrence or the date of death. The follow-up time for RFS was time until diagnosis of relapse or until death or last contact (clinical examination and/or CT/MRI scan) respectively. The end of observation period was 28 February 2021. 

### 2.3. Tracer Injection and Imaging

**Figure 1:**
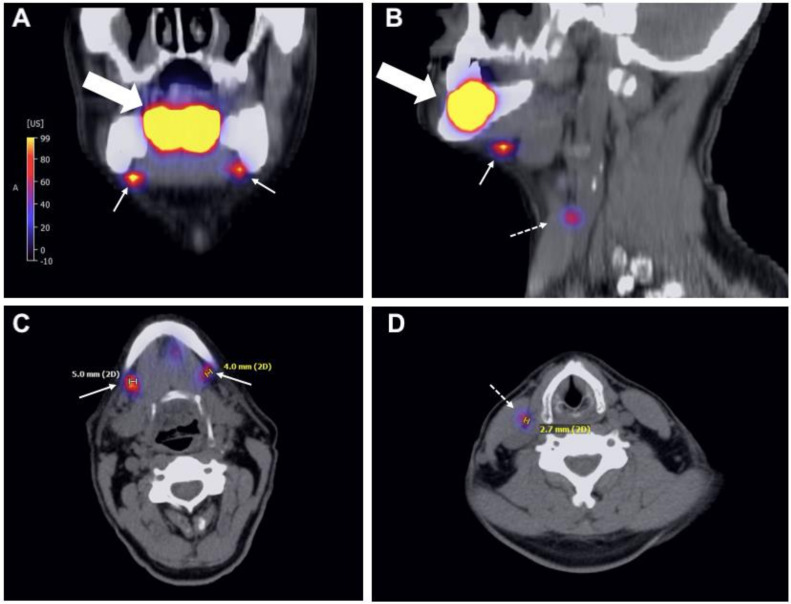
Multiplanar reconstruction of the SPECT/CT of patient no. 9: the two pictures above show the primary tumor of the floor of mouth (thick arrow) and the two foci in level Ib in the coronal (**A**) and sagittal planes (**B**). In the latter picture, a non-sentinel node downstream of the right sentinel node is marked with a thin dotted arrow. The pictures (**C**,**D**) show the two sentinel lymph nodes and one non-sentinel lymph node in the axial plane.

The tracer ^99m^technetium-(^99m^Tc)-tilmanocept was applied according to the manufacturer’s (Norgine GmbH, Wettenberg, Germany) guidelines in the Department of Nuclear Medicine, Charité-University Hospital Berlin, Germany. In brief, on the afternoon of the day before surgery tilmanocept was radiolabeled with ^99m^Tc. The following protocol was used: the patient was placed supine on the examination table of a standard clinical solid-state cadmium-zinc-telluride SPECT/CT (single photon emission computed tomography/x-ray computed tomography) camera (GE Healthcare, Discovery 670). The radiotracer 99mTc-tilmanocept was injected around the tumor’s center. Immediately after injection, dynamic planar lymphoscintigraphy was performed in anterior and lateral projections using the L-mode (matrix size 128 × 128, field of view (FOV) 566 × 566 mm) acquiring 20 images per projection over 10 min. Static planar imaging in anterior and lateral projection was conducted (matrix 256 × 256, FOV 566 × 566 mm, and acquisition time 5 min), followed by a SPECT/low-dose-CT of the head and neck region (emission tomography: matrix 128 × 128, pixel size 4.9 mm, FOV 630 × 630 mm, low dose CT: matrix 512 × 512, FOV 500 × 500 mm, 3.75 mm slice thickness, tube current 120 kV, and 29 mAs). Multiplanar reconstruction of SPECT/low-dose CT was performed in axial, coronal and sagittal reconstructions. An imaging example is displayed in Figure 1. Sentinel lymph nodes (SLNs) were determined as the first focus during imaging and there was no maximum number of activity sides determined as SLNs.

### 2.4. Surgical Procedure

Surgery was performed on the morning of the next day under general anesthesia. The maximum time interval between injection and surgery was 24 h. In 11 out of 13 cases, resection of the primary tumor was carried out firstly, in order to reduce background radiation. Secondly, SLNB was performed. The locations of sentinel nodes were roughly described by radioactive labeling, whereby the exact anatomical position had to be detected with a portable gamma probe (C Track Galaxy System, Care Wise Medical Products, Tampa, FL, USA). After identification, lymph nodes were extirpated and the signal was also confirmed after removal ex vivo. Consecutively, signaling of the neck was double checked to assure SLNs were resected accordingly. Detected lymph nodes were collected and immediately fixed with formalin for further pathological analysis.

### 2.5. Statistical Analysis

The data were collected in Microsoft Excel (v.16; Microsoft Corporation, Redmond, WA, USA) and analyzed by using IBM^®^ SPSS^®^ for Mac (v.27.0; IBM Corp., New York, NY, USA).

## 3. Results

The study consisted of 13 patients (6 men and 7 women; mean age: 65.7 years, ranging from 47 to 89 years). Most of the patients suffered from an OSCC of the floor of mouth (*n* = 6), followed by tongue (*n* = 5) and upper alveolar crest/hard palate (*n* = 2) (Table 1). None of the patients had any clinical or radiological signs of lymph node metastasis. 

All patients were treated with primary radical tumor resection and SLNB using ^99m^Tc-tilmanocept in a two-day protocol. In all cases, SLNs were successfully identified with a range of 1 to 7 in number and surgically removed. No unexpected bilateral drainage from a lateral carcinoma (no contact to or exceeding the midline) could be observed. However, in one case, bilateral drainage was seen in a patient with OSCC of the floor of mouth located on the right side. The lesion was located close to the midline but did not cross it (Table 1; case no. 9). 

The mean duration of surgical intervention including primary tumor resection, was 121 min (range: 74–233 min). None of the patients required microvascular tissue transfer to reconstruct the defect after primary tumor resection. The average length of hospital stay was 4.7 days (range: 3–8 days). Two out of three resected sentinel lymph nodes were positive (SLN+) in one patient (Table 1; case no. 12), who suffered from an OSCC of the tongue. This resulted in a modified radical neck dissection (MRND) on the affected side in a second operation (one more metastasis was found). All patients with negative SLNB had no further surgical therapy and received follow-up examinations including clinical examinations and CT or MRI scans, according to current guidelines.

The mean follow-up for all sentinel lymph node negative (SLN-) patients (*n* = 12) was 20.3 months (range: 10–28 months). No local or regional/distant (nodal) recurrences were observed and none of the patients died (RFS = 100%). Table 1 summarizes the clinical, radiological and histopathological characteristics of the cohort. 

Figure 1 shows (exemplarily) the multiplanar reconstruction of the SPECT/CT of patient no. 9 with two sentinel lymph nodes in level Ib.

## 4. Discussion

Worldwide, there is no consistent agreement as to how to proceed in cases of cN0 necks in OSCC. Although there is a recommendation for END in cases with a high likelihood of occult lymph node metastases, there are different approaches for low-risk patients with early-stage of OSCC, including END, SLNB or watchful waiting [3]. 

Recent data have shown the benefit of END at the time of primary surgery as compared to watchful waiting followed by therapeutic neck dissection in case of nodal relapse in patients with early-stage OSCC [5]. However, since there are only about 30% occult metastases [4,5], it calls into question the general use of END in this cohort, leading to morbidity without benefit in about 70% of patients. This connection causes more countries to allow surgical de-escalation in terms of SLNB in their guidelines [6].

Based on the convincing results published in numerous studies worldwide, we perform SLNB in our department to avoid neck dissection in patients diagnosed with early-stage OSCC. This study presents a protocol for SLNB using ^99m^Tc-tilmanocept, a comparatively new tracer. Furthermore, despite its limitations (including the low number of cases and the limited follow-up period), this study confirms that SLNB using ^99m^Tc-tilmanocept is a reliable and safe procedure for staging early-stage, clinically node-negative OSCC. Only a few studies exist that describe SLNB with ^99m^Tc-tilmanocept in OSCC [17,18,19,20].

There are different tracers clinically available. ^99m^Tc-nano-colloid and ^99m^Tc-sulfur-colloid are well established colloid tracers [14]. New tracers, such as ^99m^Tc-tilmanocept could potentially replace conventional tracers. Patho-physiologically, ^99m^Tc-tilmanocept is taken up into (lymph node) macrophages by binding to the CD206 mannose receptor. This enables a rapid clearance at the injection site, so that there is a more reliable detection of lymph nodes [6,16]. A recent study published by den Toom et al. in 2020 showed higher clearance at the injection site of ^99m^Tc-tilmanocept as compared to ^99m^Tc-nanocolloid in OSCC [17]. Due to these properties, ^99m^Tc-tilmanocept seems to diminish the “shine-trough effect”, which describes difficulties of differentiation between the injection site of the radiotracer and actual lymph node, in contrast to colloid tracers that persist at the injection site for a longer time [18]. 

These technical properties are reflected by other studies. A previous study using ^99m^Tc-sulfur colloid as the tracer analyzed the outcome of conventional SLNB combined with END and revealed a false negative rate of 9.8% in their patient cohort [21]. In contrast, another study using ^99m^Tc-tilmanocept observed a reduction of false negative findings to 2.56% [18], demonstrating better results. However, the difference between ^99m^Tc-tilmanocept and ^99m^Tc-sulfur colloid has been part of a randomized, blinded clinical trial in breast cancer [22]. This study could not find any differences between these tracers. In a case series of melanoma, no significant differences could be found between ^99m^Tc-sulfur colloid and ^99m^Tc-tilmanocept [23]. However, significantly lower radiation dosages, shorter mapping times and decreased numbers of sentinel nodes were removed in patients treated with ^99m^Tc-tilmanocept as compared to ^99m^Tc-sulfur colloids [23].

In this study, the floor of the mouth was the most frequent tumor location. The identification of sentinel lymph nodes for this site is generally considered as challenging [9,10,24]. Problems are associated with difficulties of differentiation between the injection site of the radiotracer and lymph nodes in close proximity in level I [9]. The protocol for SLNB presented in this study allowed for the identification of sentinel lymph nodes in all patients. For floor of mouth tumors, sentinel lymph nodes could be detected accurately and displayed free of superimposition. Of these, no locoregional recurrence occurred after a mean follow-up of 21.2 months.

In one patient in this study (patient no. 9), besides picking all three desired sentinel nodes, there were also four non-sentinel nodes resected within the same surgery due to close contact. All sentinel nodes showed a diameter of 4–6 mm, whereas the diameters of the non-sentinel nodes were larger (7–10 mm). The smaller sizes of sentinel lymph nodes in this patient suggest that these would have possibly been missed in the pathological analysis of a complete END. Consequently, one could hypothesize that SLNB is able to detect occult metastases in small lymphnodes and can therefore be considered as a safe staging tool. However, all large multi-center studies reveal a sensitivity of SLNB below 100%. According to Liu et al., its pooled sensitivity is around 0.87 [11]. 

Reasons for imperfect sensitivity are unclear but may be associated with surgeons feeling unconfident with the new technique. Alkureishi et al. discussed a learning curve when first introducing SLNB to medical units [9]. Certainly, injection has to be performed by an experienced nuclear radiologist and surgical departments have to address the time interval between injection and surgery when scheduling the operation. SLNB has been introduced as a new technique to our department, especially for patients with skin cancer. The results of this study illustrate that departments may succeed, even if the technique is newly established and extended to the oral cavity as challenging area. Another reason for a certain inaccuracy of SLNB might be associated with abnormal lymph drainage patterns [25]. 

However, the latter might also be regarded as an advantage of SLNB and the presented imaging protocol using SPECT/CT, allowing safe identification of the SLN localization. Abnormal lymph drainage patterns might not be identified with END [26]. In this study, in many cases, there was more than one focus tagged by the marker, which indicates that individual drainage patterns are possible. However, we did not observe unexpected contralateral/bilateral drainage from a lateral carcinoma. In one case, bilateral drainage was seen in a patient with OSCC of the floor of mouth located on the right side. However, the lesion was located close to the midline but did not cross it. A recent prospective study comparing SLNB and END found a rate of 6.8% for the contralateral SLN (signal) [27]. Other studies reported rates of 23% [28] and 12% [8], respectively. These patients will presumably benefit especially from SLNB, since during an ipsilateral neck dissection (according to current guidelines), these contralateral lymph nodes are not harvested and examined.

There is common agreement that SLNB, as a less invasive operation than END, offers better functional outcomes. Fewer scars and swallowing problems as well as less sensory and shoulder dysfunction, were the main advantages of SLNB [29,30,31]. However, most of these studies specifically compare SLNB versus END. Therefore, possible second operations after positive SLNB are not taken into account, which might diminish the predominance of SLNB in terms of morbidity to some extent.

The role of positron emission tomography (PET) in combination with a CT scan (PET/CT) as staging procedure and their value in the prediction of cervical lymph node metastases has been a matter of debate. According to the current German guideline, ^18^F-FDG-PET can increase diagnostic specificity and sensitivity of cervical lymph node staging when combined with CT/MRI. The value of PET and PET/CT in primary staging of OSCC has been evaluated in several studies. A prospective study by Pentenero et al. published in 2008 showed an accuracy of 66.7%, a specificity of 76.9% and a negative predictive value of 83.3% for the use of ^18^F-FDG-PET/CT for detection of nodal metastases in OSCC [32]. Liao et al. evaluated the accuracy of ^18^F-FDG PET in a cohort of 473 patients with OSCC. The patient-based sensitivity and specificity were 77.7% and 58.0% for the detection of neck metastases [33]. Ng et al. found a significantly higher sensitivity of ^18^F-FDG PET for the detection of cervical nodal metastasis on a level-by-level basis compared to CT/MRI (74.7% vs. 52.6%), whereas their specificities appeared to be similar (93.0% vs. 94.5%) [34]. According to those findings, PET/CT may be considered as a useful addition in the staging process for OSCC. However, clinical application of PET/CT in the cN0 neck seems to be limited due to the suboptimal sensitivity for small metastases <10 mm and the comparatively high number of false-positive findings [32,35,36]. With a pooled sensitivity of 0.87 and a negative predictive value of 0.94 [11], SLNB seems to be superior in comparison to PET/CT. Comparing different diagnostic tools including PET and SLNB in a large meta-analysis, Liao et al. found that the SLNB procedure has the best diagnostic performance for cN0 head and neck cancer [37]. However, in cases of unclear staging using CT/MRI, additional PET/CT may provide further information as basis for the decision-making for SLNB/END vs. therapeutic neck dissection.

## 5. Conclusions

This single-center study presents a protocol for SNLB using ^99m^Tc-tilmanocept. Herewith, this study corroborates previous large multi-center studies concluding that SLNB represents a safe and reliable staging method for patients suffering from early-stage, clinically node-negative OSCC. SLNB using ^99m^Tc-tilmanocept has proved to be a reliable and safe staging method for these patients in our cohort. SLNs could be detected accurately and displayed free of superimposition. Their possible superiority to colloids has to be evaluated in further studies. Advantages concerning possible identification of micro-metastases and contralateral metastases, as well as lower morbidity and costs compared to END, might give SLNB a key role in early-stage OSCC in the future. Large multicenter prospective randomized studies are needed for a complete paradigm shift in the real clinical situation in Germany.

## Figures and Tables

**Table 1:** Clinical, radiological and histopathological characteristics of the patients.

No	Age	Gender	Tumor Localization	Side	cTNM	Number of Foci Detected in SPECT/CT	Side of Foci Detected	Number of Sentinel Nodes Resected	Number of Positive Sentinel Nodes	Duration of Surgical Procedure (Minutes)	Length of Hospital Stay (Days)	pTNM	Follow-Up (Months)	Locoregional Recurrence
1	56	male	tongue	right	T1N0M0	3	right	7	0	141	3	T1N0	28	no
2	79	female	hard palate	right	T1N0M0 *	1	right	1	0	83	4	T1N0	26	no
3	67	female	floor of mouth	left	T1N0M0	3	left	1	0	147	6	T1N0	28	no
4	47	female	floor of mouth	left	T1N0M0	1	left	4 ****	0	233	7	T1N0	24	no
5	61	female	floor of mouth	left	T1N0M0 **	1	left	2	0	82	6	T1N0	20	no
6	78	female	floor of mouth	right	T1N0M0	2	right	2	0	149	4	T1N0	24	no ***
7	65	male	floor of mouth	both	T1N0M0	2	both	3	0	138	4	T2N0	20	no
8	81	female	tongue	left	T1N0M0	1	left	2	0	145	8	T1N0	25	no
9	58	male	floor of mouth	right	T1N0M0	2	both	3	0	80	3	T1N0	11	no
10	53	male	tongue	right	T1N0M0	1	right	2	0	95	4	T1N0	15	no
11	89	male	Upper alveolar crest/hard palate	left	T1N0M0	1	left	1	0	74	4	T4aN0	13	no
**12**	**58**	**female**	**tongue**	**left**	**T1N0M0**	**1**	**left**	**3**	**2**	**105**	**4**	**T1N2b**	**11**	**no**
13	62	male	tongue	left	T1-T2N0M0	1	left	4	0	104	4	T1N0	10	no

* During the staging process a lesion within the right breast was found, which was diagnosed as fibroadenoma in the further clinical course. ** During the staging process a lesion within the thoracic spine was found, which was considered as benign after PET-CT. *** During last CT-scan an asymmetric base of tongue on the left side was noted. The patient refused a biopsy due to the absence of clinical symptoms. **** Beside the focus detected in SPECT/CT, which was considered to be the sentinel lymph node, three additional foci were removed. Patient no. 12 is indicated in bolt due to the positive sentinel nodes.

## Data Availability

The data presented in this study are available on request from the corresponding author.
